# Decision Aids for Prostate Cancer Screening Choice: A Systematic Review and Meta-analysis

**DOI:** 10.1001/jamainternmed.2019.0763

**Published:** 2019-06-24

**Authors:** Jarno M. Riikonen, Gordon H. Guyatt, Tuomas P. Kilpeläinen, Samantha Craigie, Arnav Agarwal, Thomas Agoritsas, Rachel Couban, Philipp Dahm, Petrus Järvinen, Victor Montori, Nicholas Power, Patrick O. Richard, Jarno Rutanen, Henrikki Santti, Thomas Tailly, Philippe D. Violette, Qi Zhou, Kari A. O. Tikkinen

**Affiliations:** 1 Department of Urology, Tampere University Hospital, Tampere, Finland; 2 Faculty of Medicine and Life Science, University of Tampere, Tampere, Finland; 3 Department of Health Research Methods, Evidence, and Impact, McMaster University, Hamilton, Ontario, Canada; 4 Department of Urology, Helsinki University Hospital and University of Helsinki, Helsinki, Finland; 5 Department of Medicine, University of Toronto, Toronto, Ontario, Canada; 6 Division of General Internal Medicine, Department of Internal Medicine, University Hospitals of Geneva, Geneva, Switzerland; 7 Department of Anesthesia, McMaster University, Hamilton, Ontario, Canada; 8 Urology Section, Minneapolis Veterans Administration Health Care System, Minneapolis, Minnesota; 9 Department of Urology, University of Minnesota, Minneapolis; 10 Knowledge and Evaluation Research Unit, Mayo Clinic, Rochester, Minnesota; 11 Division of Urology, Department of Surgery, Western University, London, Ontario, Canada; 12 Faculty of Medicine and Health Sciences, Université de Sherbrooke, Sherbrooke, Quebec, Canada; 13 Department of Internal Medicine, Tampere University Hospital, Tampere, Finland; 14 Department of Urology, Ghent University Hospital, Gent, Belgium; 15 Department of Surgery, Woodstock General Hospital, Woodstock, Ontario, Canada; 16 Department of Public Health, University of Helsinki, Helsinki, Finland

## Abstract

**Importance:**

US guidelines recommend that physicians engage in shared decision-making with men considering prostate cancer screening.

**Objective:**

To estimate the association of decision aids with decisional outcomes in prostate cancer screening.

**Data Sources:**

MEDLINE, Embase, PsycINFO, CINAHL, and Cochrane CENTRAL were searched from inception through June 19, 2018.

**Study Selection:**

Randomized trials comparing decision aids for prostate cancer screening with usual care.

**Data Extraction and Synthesis:**

Independent duplicate assessment of eligibility and risk of bias, rating of quality of the decision aids, random-effects meta-analysis, and Grading of Recommendations, Assessment, Development and Evaluations rating of the quality of evidence.

**Main Outcomes and Measures:**

Knowledge, decisional conflict, screening discussion, and screening choice.

**Results:**

Of 19 eligible trials (12 781 men), 9 adequately concealed allocation and 8 blinded outcome assessment. Of 12 decision aids with available information, only 4 reported the likelihood of a true-negative test result, and 3 presented the likelihood of false-negative test results or the next step if the screening test result was negative. Decision aids are possibly associated with improvement in knowledge (risk ratio, 1.38; 95% CI, 1.09-1.73; *I*^2^ = 67%; risk difference, 12.1; low quality), are probably associated with a small decrease in decisional conflict (mean difference on a 100-point scale, −4.19; 95% CI, −7.06 to −1.33; *I*^2^ = 75%; moderate quality), and are possibly not associated with whether physicians and patients discuss prostate cancer screening (risk ratio, 1.12; 95% CI, 0.90-1.39; *I*^2^ = 60%; low quality) or with men’s decision to undergo prostate cancer screening (risk ratio, 0.95; 95% CI, 0.88-1.03; *I*^2^ = 36%; low quality).

**Conclusions and Relevance:**

The results of this study provide moderate-quality evidence that decision aids compared with usual care are associated with a small decrease in decisional conflict and low-quality evidence that they are associated with an increase in knowledge but not with whether physicians and patients discussed prostate cancer screening or with screening choice. Results suggest that further progress in facilitating effective shared decision-making may require decision aids that not only provide education to patients but are specifically targeted to promote shared decision-making in the patient-physician encounter.

## Key Points

**Question:**

What is the association of decision aids vs usual care with shared decision-making in men deciding whether to undergo prostate cancer screening?

**Findings:**

This systematic review and meta-analysis of 19 randomized clinical trials comparing decision aids for prostate cancer screening (12 781 men) found that decision aids are probably associated with a small reduction in decisional conflict and are possibly associated with an increase in knowledge. Decision aids are possibly not associated with whether physicians and patients discuss prostate cancer screening and are possibly not associated with actual screening decisions.

**Meaning:**

Randomized clinical trials have failed to provide compelling evidence for the use of decision aids for men contemplating prostate cancer screening that have, up to now, undergone rigorous testing to determine their outcome.

## Teaser

This systematic review and meta-analysis of 19 randomized clinical trials estimates the association of decision aids with decisional outcomes in prostate cancer screening.

## Introduction

Owing to increasing use of prostate-specific antigen (PSA) screening, the incidence of early-stage prostate cancer has increased during the last 25 years.^1^ Advocates of screening often cite the European Randomized study of Screening for Prostate Cancer (ERSPC)^2^—of the available trials, the one at lowest risk of bias^3^—that suggested that PSA screening reduces prostate cancer–specific mortality but not overall mortality.^2^ Opponents of screening often cite an earlier meta-analysis^4^ or other major trials^5,6^ that reported no association between PSA screening and prostate cancer–specific mortality and point out possible harms associated with surgery or radiotherapy.^7^

Men’s choice of whether to undergo prostate cancer screening is sensitive to their values and preferences: that is, fully informed men will make different choices depending on their experience and perspective. For such decisions, shared decision-making, characterized by cooperative communication between patient and clinician in which they share knowledge, values, and preferences, represents an ideal approach to decision-making.^8^ Major guidelines therefore acknowledge the importance of informing men about the risks and benefits of PSA screening.^9,10,11,12^ The US Preventive Services Task Force has recently recommended that the decision to undergo prostate cancer screening should be an individual one in which men should discuss potential benefits and harms with their clinician before screening and recommended that men who do not express a clear preference for screening should not be screened.^11^ Even more recently, a *BMJ* Rapid Recommendations’ panel made a weak recommendation against systematic PSA screening that acknowledged the need for shared decision-making.^12^

Shared decision-making is challenging because of time constraints and the specific skills that it requires.^13^ Well-designed decision aids may, at least in part, address these challenges by summarizing the current best evidence and by supporting conversations that address the issues that matter most to patients.^14,15^ The association of decision aids with the decision-making process remains, however, uncertain.^8^ We therefore undertook a systematic review and meta-analysis of the randomized clinical trials (RCTs)—many of which were conducted before major PSA trials,^2,5,6^ such as ERSPC,^2^ were published—that have addressed the effect of decision aids on the decision-making process in the context of prostate cancer screening.

## Methods

We registered the protocol in the International Prospective Register of Systematic Reviews (PROSPERO CRD42016052816) and followed the Preferred Reporting Items for Systematic Reviews and Meta-analyses (PRISMA) reporting guideline.^16^

### Data Sources and Searches

We performed the search, developed in collaboration with an experienced research librarian (R.C.), on June 19, 2018, in MEDLINE, Embase, CINAHL, PsychINFO, and Cochrane Central Register of Controlled Trials (CENTRAL) without language limits (eAppendix 1 in the Supplement).

### Eligibility Criteria

We included RCTs conducted among men who were potentially considering undergoing prostate cancer screening that compared decision aid interventions for prostate cancer screening with usual care. We evaluated decision aids and study protocols and judged interventions as either decision aids, information material, or usual care (not overlapping categories). We defined the interventions as decision aids if the material helped men making individual choices and included information regarding the association of screening with the following patient-important outcomes: risk of dying, risk of urinary or bowel symptoms, and risk of erectile dysfunction. We defined the intervention as usual care if clinicians provided no formal, structured presentation of information and informative material if interventions provided some structured information but did not meet our definition of a decision aid (eAppendix 2 in the Supplement).

We excluded studies comparing one decision aid with another and those that did not report on any of our specified outcomes (see the Outcomes subsection). We also excluded studies in which less than 50% of participants in intervention groups used a decision aid.

### Outcomes

We evaluated the following outcomes: knowledge regarding prostate cancer screening, decisional conflict, discussions regarding screening between men and their physicians (screening discussion), decisions determining whether screening took place (actual screening decision), and satisfaction with screening decision.

### Risk of Bias and the Quality of Decision Aids

We assessed the risk of bias using a modified version of the Cochrane Collaboration risk of bias tool addressing 5 criteria (eAppendix 3 in the Supplement). For each criterion, studies were judged to be at either high or low risk of bias. Studies with a high risk of bias for 3 or more criteria were classified as being at high risk of bias overall.

We identified decision aids used in the studies by following a multistep approach: (1) we first reviewed original articles to identify links or references to electronically available decision aids or those provided as appendices; (2) if unavailable, we conducted electronic searches for decision aids online; and (3) we contacted study authors by email, requesting access to the decision aid. We evaluated the available decision aids using a modified version of the International Patient Decision Aid Standards instrument (IPDASi), version 3 for screening^17^ by assessing 10 criteria (eAppendix 4 in the Supplement). We rated each criterion as met or unmet and summed the number of criteria met.

### Study Selection and Data Extraction

We developed standardized forms with detailed instructions for screening of abstracts and full texts, risk of bias, quality of assessments of decision aids, and data extraction. Independently and in duplicate, 2 methodologically trained reviewers (J.M.R., T.P.K., S.C., A.A., P.J., N.P., P.O.R., J.R., H.S., and T.T.) applied the forms to screen study reports for eligibility and extracted data. Reviewers resolved disagreement through discussion and, if necessary, through consultation with an adjudicator (K.A.O.T.). We sent our consensus data extraction to the original authors for confirmation or correction and asked for clarification regarding missing or unclear information.

### Statistical Analysis

For continuous outcomes in which investigators used different instruments to measure a construct, we standardized scores on a range from 0 to 100^18,19^ and summarized the data as means and SDs or, for skewed distributions, medians and interquartile ranges. For continuous variables, we expressed effects as mean differences and 95% CIs and for binary outcomes, as relative risks and 95% CIs. To obtain the absolute difference, we chose the percentage correct of the median of the control groups and applied the point estimate and 95% CIs of the pooled relative risk to that value. We categorized outcome effects as short-term (effect estimated ≤1 month after decision aid use) and long-term (>1 month after decision aid use) and focused on the last time point in either period in the primary analysis. All *P* values were from 2-sided tests, and results were deemed statistically significant at *P* < .05.

We conducted meta-analyses when data for a particular outcome were available from at least 3 trials. For studies with more than 1 intervention group, if we failed to reject the null hypothesis that the intervention groups did not differ (*z* test at 5% significance level), we pooled the groups within the study; if results differed, we used only the group with the largest effect. To study the potential differences in intervention effects on the outcomes by length of follow-up (short-term defined as ≤1 month and long-term as >1 month), we first conducted the repeated measure, random-effects, weighted mixed regression model analysis. The dependent variable was the outcome mean and the independent variables were the intervention, the follow-up term, the interaction of intervention and follow-up term, the random effects in study, and the baseline data. We reported the pooled analyses separately by length of follow-up if the interaction effect was significant; if not, we reported analyses using the longest follow-up. For analyses in which the *I*^2^ statistic was greater than 0%, we pooled the results using Hartung-Knapp-Sidik-Jonkman random-effects models. If the *I*^2^ statistic was 0%, we pooled results using fixed-effects models because, under these circumstances, the fixed-effects method is superior to the Hartung-Knapp-Sidik-Jonkman method in type I error.^20^ We examined the following variables as potential sources of heterogeneity using meta-regression: allocation concealment, blinding of data collectors, and missing data (low vs high risk of bias for all variables). We hypothesized that effects would be larger in high-risk-of-bias trials.

### Quality of Evidence

To assess the quality of evidence, we used the Grading of Recommendations, Assessment, Development and Evaluations (GRADE) approach that classifies evidence as high, moderate, low, or very low quality.^21^ We used published GRADE guidance for ratings of risk of bias,^22^ consistency,^23^ directness,^24^ precision,^25^ and publication bias.^26^ We made 1 major modification of GRADE: the GRADE quality of evidence ratings are intended to address causal inferences; because of journal policy, we applied the quality ratings to issues of association.

## Results

**Figure 1.**
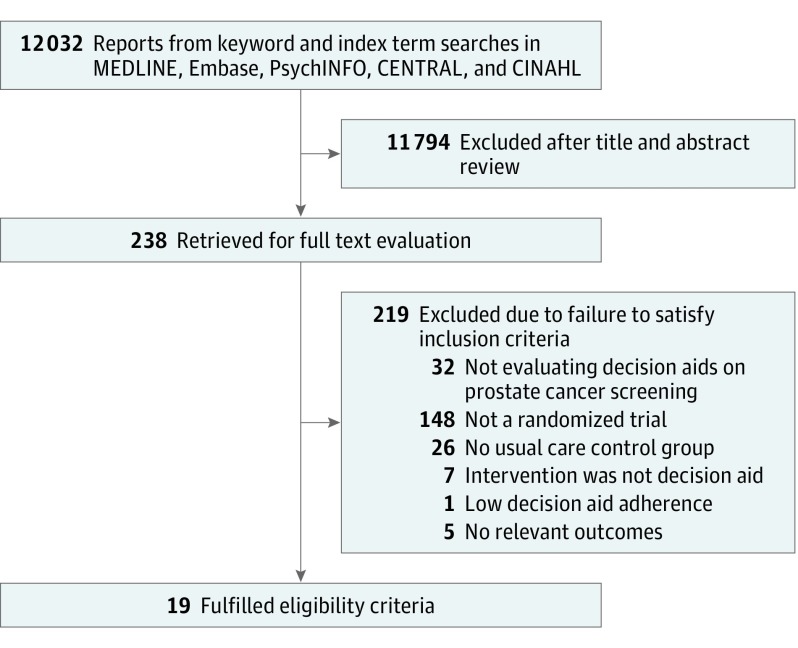
Flowchart Outlining the Literature Search and Article Evaluation Process

Of 12 032 potentially relevant reports, 238 proved potentially eligible; after full-text screening, 19 articles^27,28,29,30,31,32,33,34,35,36,37,38,39,40,41,42,43,44,45^ proved eligible (Figure 1). Six of the 19 authors (32%) confirmed the accuracy of our data extraction^28,33,37,39,42,43^; none corrected errors or added additional information. Eleven of the 19 authors (58%)^27,29,30,32,34,35,38,40,41,44,45^ could not be contacted, and 2 authors (11%)^31,36^ were unable to assist. Trials were published between 1999 and 2017 (eFigure 1 in the Supplement) and randomized 12 781 men; the median of mean ages was 59 years (interquartile range, 57-62 years). Sixteen studies were performed in the United States, 2 in the United Kingdom, and 1 in Canada (Table 1).

**Table 1.** General Characteristics, Overall Risk of Bias, and IPDASi Evaluation of Included Randomized Trials

Source	Country	Men Randomized, No.	Decision Aid Groups			Control Groups			Recruitment Years	Overall Risk of Bias	IPDASi Score
			Type of Intervention	Men Randomized, No.	Age, y	Type of Control	Men Randomized, No.	Age, y			
Stamm et al,^27^ 2017	United States	329	Group 1: printed leaflet; group 2: printed leaflet and shared decision-making	Group 1: 113; group 2: 110	Group 1: 62; group 2: 61	None	106	63	NR	High	NA
Landrey et al,^28^ 2013	United States	303	Printed leaflet	145	62	None	158	62	2009-2010	High	2
Taylor et al,^29^ 2013	United States	1893	Group 1: printed booklet; group 2: computer based	Group 1: 630; group 2: 631	57	None	632	57	2007-2010	High	9
Lepore et al,^30^ 2012	United States	490	Telephone education and printed booklet	244	55	Food information	246	55	2005-2006	High	2
Sheridan et al,^31^ 2012	United States	130	Video, printed leaflet, and individual education	60	57	Highway safety video	70	58	2005-2006	Low	4
Chan et al,^33^ 2011^a^	United States	321	Group education: video, printed booklet, script, and slides	160	NR	Group education: diabetes video and discussion	157	61	NR	Low	NA
Allen et al,^32^ 2010^a^	United States	2615	Computer based	1118	NR	None	1497	NR	2006-2007	High	NA
Evans et al,^34^ 2010	United Kingdom	514	Group 1: computer based; group 2: printed booklet	Group 1: 129; group 2: 126	NR	None	259	NR	2008	High	10
Rubel et al,^35^ 2010	United States	200	Printed booklet	100	59	None	100	59	2005	Low	NA
Frosch et al,^36^ 2008	United States	611	Group 1: computer-based chronic disease trajectory model; group 2: computer-based traditional model; group 3: computer-based combination of both 1 and 2	Group 1: 153; group 2: 155; group 3: 152	Group 1: 58; group 2: 59; group 3: 59	Link to websites	151	59	2005-2006	High	NA
Husaini et al,^37^ 2008^a^	United States	430	Group education: video, printed leaflet, and teaching session	235	55	None	115	50	NR	High	NA
Stephens et al,^38^ 2008	United States	440	Printed booklet	200	NR	None	200	NR	NR	Low	7
Krist et al,^39^ 2007	United States	497	Group 1: printed booklet; group 2: computer based	Group 1: 196; group 2: 226	Group 1: 57; group 2: 56	None	75	57	2002-2004	High	9
Taylor et al,^40^ 2006	United States	294	Group 1: video; group 2: printed booklet	Group 1: 95; group 2: 98	Group 1: 56; group 2: 57	None	92	55	2001-2002	High	2
Watson et al,^41^ 2006	United Kingdom	1960	Printed leaflet	980	59	None	980	59	2004	High	7
Partin et al,^42^ 2004	United States	1152	Group 1: printed booklet; group 2: video	Group 1: 384; group 2: 384	68	None	384	68	2001	High	4 (printed booklet); 6 (video)
Wilt et al,^43^ 2001	United States	342	Printed leaflet	163	73	None	179	70	1998	Low	5
Davison et al,^44^ 1999	Canada	100	Individual education: verbal and printed	50	64	General medical information	50	61	NR	High	NA
Volk et al,^45^ 1999	United States	160	Video and printed leaflet	80	59	None	80	60	1997	High	6

Abbreviations: IPDASi, International Patient Decision Aid Standards instrument, version 3; NA, not applicable; NR, not reported.

^a^ Cluster randomized trial.

### Risk of Bias

In all 19 studies, the allocation sequence was adequately generated; in 9 studies (47%), allocation was adequately concealed; and in 8 studies (42%), data collectors were blinded. Missing data were judged as high risk of bias in 7 of 13 studies (54%) for actual screening decision and in 11 of 19 studies (58%) for other outcomes (knowledge, screening discussion, decisional conflict, and satisfaction with decision) (Table 1; eFigure 2 in the Supplement).

### Decision Aids

Investigators used several types of decision aids: 13 of 19 studies used printed material (8 used booklets of 8-28 pages^29,30,34,35,38,39,40,42^ and 5 used leaflets of 1-2 pages^27,28,41,43,45^), 5 studies used education (2 used group sessions^33,37^ and 3 used individual education^30,31,44^), 5 studies used computer-based tools,^29,32,34,36,39^ and 4 studies used videos.^31,40,42,45^ Two studies used the same video.^42,45^ One study used shared decision-making^27^ (eTable 1 in the Supplement).

We identified 12 decision aids: 5 by reviewing original articles,^28,30,31,41,43^ 4 by electronic searches,^29,34,38,40^ and 3 from the authors.^39,42,45^ Two authors reported that the decision aid was no longer available (eTable 1 in the Supplement).^35,44^ Three decision aids scored well (8-10 points out of 10), 4 scored less well (5-7 points), and 5 scored poorly (≤4 points); the overall IPDASi mean (SD) score was 5.6 (2.9) (range, 2-10). All decision aids reported the screening aim; 11 of 12 decision aids (92%) reported the association of screening with overall or prostate cancer–specific mortality; and 10 of 12 decision aids (83%) reported the harms of the increase in surgery and radiotherapy that accompanies the increased diagnosis of prostate cancer consequent to screening (erectile dysfunction, urinary incontinence, and bowel problems). Four of 12 decision aids (33%) presented information regarding the probability of having a true-negative result; 3 of 12 decision aids (25%) presented the probability of a false-negative result or the next step if screening results were negative. Two of 12 decision aids (17%) presented the likelihood of detecting prostate cancer with and without the use of screening (eFigure 3 in the Supplement).

### Outcomes

#### Knowledge

**Figure 2.**
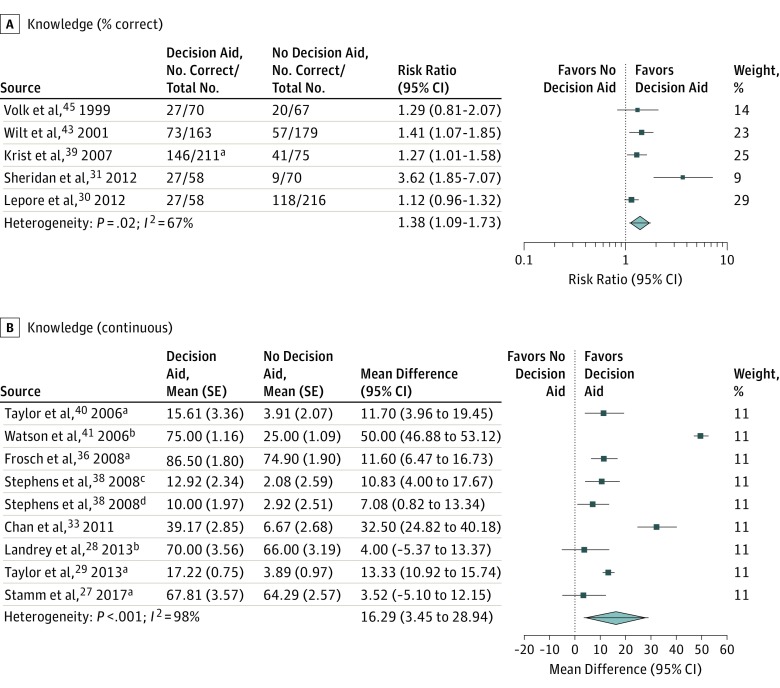
Forest Plots of Short-term Prostate Cancer Screening Knowledge ^a^Pooled result from multiple groups. ^b^Unadjusted from baseline. ^c^African American study population. ^d^Non–African American study population.

Of the 13 studies reporting short-term knowledge, 8 reported data as a continuous variable and 5 reported the proportion of correct items. Because the SDs of the latter are much smaller (owing to the nature of binomial distribution), they would dominate a pooled result of all 13 studies; therefore, we analyzed them separately. Pooled estimates from 8 studies reporting data as a continuous variable showed an increase in knowledge for decision aids (mean difference, 16.29; 95% CI, 3.45-28.94; low-quality evidence; Table 2 and Figure 2B). The proportion of correctness data from 5 studies demonstrated improved knowledge with decision aids, although the 95% CI includes a very small and likely unimportant difference (risk ratio, 1.38; 95% CI, 1.09-1.73; risk difference, 12.1; low-quality evidence; Table 2 and Figure 2A). Studies failed to demonstrate an association with knowledge in the long term (mean difference, 5.47; 95% CI, −0.52 to 11.45; low-quality evidence; eFigure 4 in the Supplement).

**Table 2.** GRADE Evidence Profile: Decision Aid vs Usual Care for Prostate Cancer Screening

Quality Assessment						Summary of Findings		
No. of Patients With Data (No. of Studies)	Risk of Bias	Inconsistency	Indirectness	Imprecision	Publication Bias	Relative Effect (95% CI)	Absolute Difference (95% CI)	Certainty in Estimates
**Knowledge (short-term; percentage correct)**								
1167 (5)	Serious limitations^a^	No serious limitations	No serious limitations	Serious limitations: CI includes a very small and likely unimportant difference	Undetected	Decision aid increased discussion about prostate cancer screening by 38% (from 9% to 73% increase)	Mean difference of 12.1 (from 2.9 increase to 24.5 increase) on percentage correct favoring decision aid	Low^b^
**Knowledge (short-term; continuous)**								
4272 (8)	Serious limitations^c^	No serious limitations	No serious limitations	Serious limitations: CI includes a very small and likely unimportant difference	Undetected	NA	Mean difference of 16.3 (from 3.5 increase to 28.9 increase) on 100-point scale favoring decision aid	Low^b^
**Decisional Conflict**								
3700 (6)	Serious limitations^d^	No serious limitations	No serious limitations	No serious limitations	Undetected	NA	Mean difference of 4.2 (from 1.3 to 7.1) on 100-point scale favoring decision aid	Moderate^e^
**Screening Discussion**								
1927 (6)	Serious limitations^f^	No serious limitations	No serious limitations	Serious limitations: CI crosses no difference	Undetected	Decision aid increased screening discussion by 12% (from 10% decrease to 39% increase)	No significant effect	Low^b^
**Actual Screening Decision**								
4286 (13)	Serious limitations^g^	No serious limitations	No serious limitations	Serious limitations: CI crosses no difference	Undetected	Decision aid decreased screening by 5% (from 13% decrease to 4% increase)	No significant effect	Low^b^

Abbreviations: GRADE, Grading of Recommendations, Assessment, Development and Evaluations; NA, not applicable.

^a^ Of the 5 studies, 3 (60%) were at high risk of bias, and 2 (40%) were at low risk of bias (Table 1; eFigure 2 in the Supplement).

^b^ The low quality of the rating reflects concerns in 3 domains: risk of bias, inconsistency, and imprecision.

^c^ Of the 8 studies, 6 (75%) were at high risk of bias, and 2 (25%) were at low risk of bias (Table 1; eFigure 2 in the Supplement).

^d^ Of the 6 studies, 5 (83%) were at high risk of bias, and 1 (17%) was at low risk of bias (Table 1; eFigure 2 in the Supplement).

^e^ The moderate quality of rating reflects concerns in 2 domains: risk of bias and imprecision.

^f^ Of the 6 studies, 4 (67%) were at high risk of bias, and 2 (33%) were at low risk of bias (Table 1; eFigure 2 in the Supplement).

^g^ Of the 13 studies, 11 (85%) were at high risk of bias, and 2 (15%) were at low risk of bias (Table 1; eFigure 2 in the Supplement).

#### Decisional Conflict

**Figure 3.**
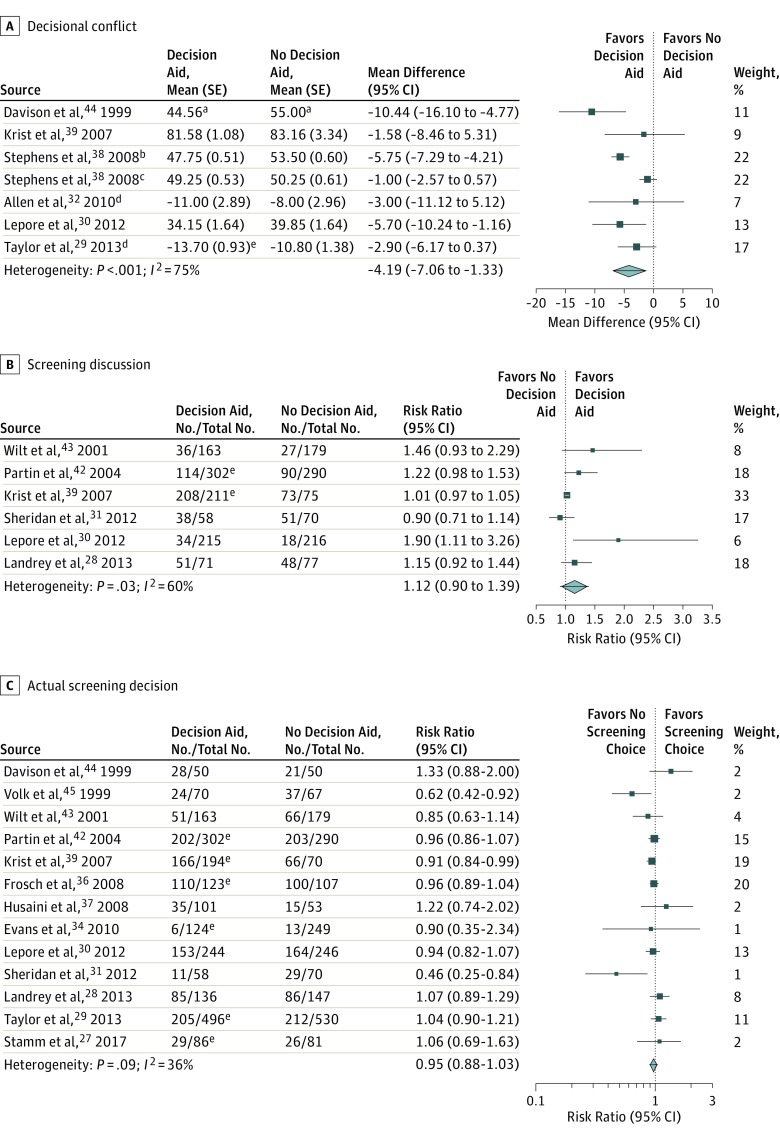
Forest Plots of Prostate Cancer Screening Decisional Conflict, Screening Discussion, and Actual Screening Decision ^a^Scaled to 100. ^b^African American study population. ^c^Non–African American study population. ^d^Pooled result from multiple groups. ^e^Unadjusted from baseline.

In the pooled analysis (6 studies), the decision aids were associated with a small but consistent and statistically significant decrease in decisional conflict (mean difference on a 100-point scale, −4.19; 95% CI, −7.06 to −1.33; moderate-quality evidence; Table 2 and Figure 3A).

#### Screening Discussion

The frequency with which a screening discussion with the clinician took place varied from 8% to 97% (median, 47%) in usual care groups and from 16% to 99% in decision aid groups (median, 52%). The pooled analysis from 6 studies failed to demonstrate an association with whether physicians and patients discussed prostate cancer screening (risk ratio, 1.12; 95% CI, 0.90-1.39; low-quality evidence; Table 2 and Figure 3B). In 4 studies,^28,39,42,43^ the decision aid was distributed 1 to 2 weeks before the visit or assessment; in 1 study,^31^ the decision aid was distributed 1 hour before the assessment; and in 1 study,^30^ the decision aid was distributed 8 months before the visit.

#### Actual Screening Decision

The frequency with which men choose to undergo prostate cancer screening ranged from 5% to 94% (median, 49%) in usual care groups and 5% to 90% in decision aid groups (median, 49%). The pooled analysis from 13 studies demonstrated no association in men’s decision to undergo or not undergo prostate cancer screening between the decision aid and usual care groups (risk ratio, 0.95; 95% CI, 0.88-1.03; low-quality evidence; Table 2 and Figure 3C).

#### Satisfaction With Decision

Three studies^29,40,45^ reported men’s satisfaction with their decision; 2 of these studies used the Satisfaction with Decision Scale,^29,45,46^ and 1^40^ used a Likert scale. Two studies reported no difference in satisfaction between the intervention and control groups.^40,46^ One study^29^ reported that men in both the group that received a printed decision aid (odds ratio [OR], 1.79; 95% CI, 1.41-2.29) and the group that received a web-based decision aid (OR, 1.29; 95% CI, 1.02-1.66) were more likely to report high satisfaction at 1 month of follow-up compared with usual care (high satisfaction reported by 60.4% in the printed decision aid group and 52.2% in the web decision aid group compared with 45.5% in the control group). This difference persisted compared with the usual care group for the printed decision aid group (OR, 1.29; 95% CI, 1.01-1.66) but not for the web-based decision aid group (OR, 1.04; 95% CI, 0.81-1.34) at 13 months of follow-up. Furthermore, participants with printed material reported significantly greater satisfaction than with web material at 1 month (OR, 1.38; 95% CI, 1.07-1.77) but not at 13 months (OR, 1.24; 95% CI, 0.96-1.60). None of these studies examined whether satisfaction varied by whether the decision was to undergo prostate cancer screening or not to undergo screening. For no outcome did risk of bias explain the variability in results (eTable 2 in the Supplement).

## Discussion

### Main Findings

To examine the association of prostate cancer screening decision aids with decisional outcomes and screening decisions, we pooled data from 19 trials. Low-quality evidence suggests that decision aids are associated with an improvement in men’s knowledge regarding prostate cancer screening, and moderate-quality evidence suggests that decision aids are associated with a small decrease in decisional conflict. Overall, decision aids proved to not be statistically significantly associated with whether physicians and patients discussed prostate cancer screening, or with men’s decision to undergo or not undergo screening (low-quality evidence). The decision aids used in these studies provided most of the crucial information (benefits and harms of screening) but typically omitted test properties of the screening tests.

### Strengths and Limitations of the Study

Strengths of our study include a comprehensive search, duplicate assessment of eligibility and data extraction, appraisal of risk of bias, use of outcomes that are important to patients, and evaluation of decision aids using the IPDASi instrument. To increase the precision of estimates, whenever possible, we conducted meta-analyses using appropriate statistical methods. The GRADE approach was applied to assess the quality of evidence for each outcome (Table 2).

Limitations of our review are largely those of the available literature. First, we were not able to use all studies: in 26 studies, there was no usual care control group, 5 studies did not report on any of our outcomes, and 1 study had very low adherence to the decision aid (eTable 3 in the Supplement). Second, we were able to conduct IPDASi evaluation in only 12 decision aids used in 13 studies. Third, most trials were performed before major PSA trials—ERSPC^2^; Prostate, Lung, Colorectal, and Ovarian Cancer Screening Trial^5^; and Cluster Randomized Trial of PSA Testing for Prostate Cancer^6^—provided data (eFigure 1 in the Supplement). Fourth, different instruments were used for assessment of knowledge. Fifth, we found only low-quality evidence for the association of decision aids with knowledge, whether a screening discussion was conducted, or patients’ decisions whether to undergo screening. Furthermore, many available decision aids have not undergone formal testing in randomized trials.

### Association With Other Studies

Three previous systematic reviews have investigated decision aids for prostate cancer screening.^47,48,49^ One review published more than 10 years ago addressed different questions and did not include 14 studies included in our review.^47^

A systematic review published in 2015 concluded that decision aids increase patient knowledge and confidence in decision-making regarding prostate cancer testing.^48^ This review included 13 studies, of which we did not include 6 studies^50,51,52,53,54,55^ because of the lack of a standard care control group, but it failed to include 12 trials that proved to be eligible in our systematic review: 11 RCTs of decision aids that were reported before the publication of their review and apparently met their eligibility criteria^28,35,36,37,38,39,40,41,43,44,45^ and one study^27^ that was published after their review appeared. The authors failed to conduct a meta-analysis.^48^

Ivlev and colleagues^49^ have published the most recent systematic review on prostate cancer screening patient decision aids and concluded that integration of decision aids in clinical practice may result in a decrease in the number of men who elect to undergo PSA testing, which may in turn reduce screening uptake. Support for this statement came from an analysis of intent to screen (risk ratio, 0.88; 95% CI, 0.81-0.95). Their meta-analysis of 2 RCTs that addressed men’s actual decision found, however, no difference between the decision aid and usual care groups (risk ratio, 0.92; 95% CI, 0.62-1.36) and is consistent with our analysis of 13 RCTs (risk ratio, 0.95; 95% CI, 0.88-1.03).

The review by Ivlev et al^49^ included 13 RCTs and 5 observational studies; to avoid bias associated with prognostic imbalance, we restricted our eligible studies to RCTs. Of the RCTs that Ivlev and colleagues^49^ included, we did not include 3 studies^54,55,56^ because they did not have a standard care control group and 1 study^57^ because it lacked our prespecified outcomes. The review by Ivlev et al^49^ failed to include 10 of our 19 eligible trials: 3 trials^28,31,33^ were considered—contrary to our judgment—as not having a decision aid group, 3 trials^29,35,38^ were excluded because they did not meet their eligibility criteria of reporting immediate or deferred intention or utilization data, 1 trial^44^ was excluded without explanation, and 3 trials^37,40,43^ were either not identified by their search or were excluded during title and abstract screening (not possible to distinguish which reason). Other differences included our measuring of screening discussions and reporting a meta-analysis of decisional conflict, which were not in the review by Ivlev et al.^49^ Ivlev and colleagues^49^ stated in their methods (including PROSPERO CRD42017060606) that they used the GRADE approach^21^; however, they provided evidence quality for only 2 outcomes: intention to undergo PSA testing and knowledge. Our judgments applying the GRADE approach^21^ included all outcomes and differed from the review by Ivlev et al^49^ regarding knowledge because we considered the failure to use blinded assessments as a reason to rate the quality of evidence downward and they did not.

### Implications for Clinicians and Policymakers, and Future Directions

Our results suggest modest and uncertain associations between existing decision aids and key outcomes: a possible increase in knowledge and likely a small decrease in decisional conflict but no apparent association with whether physicians and patients discussed prostate cancer screening or with men’s decision to undergo or not undergo prostate cancer screening. Many prostate cancer screening decision aids are available online, but only a few have undergone formal testing. All decisions aids included in our review provided education to patients, and all but 1 decision aid^27^ failed to show clear facilitation of screening discussions (ie, shared decision-making).^14^ The results demonstrate a lack of prostate cancer decision aids specifically geared toward or successful in facilitating shared decision-making.

The best available evidence suggests that PSA screening may have a small, although uncertain, benefit on prostate cancer mortality.^3^ Evidence shows, however, that PSA screening also harms men because of false-positive test results and overdiagnosis and overtreatment of prostate cancer.^3^ Before the major prostate cancer screening trials reported their results,^2,5,6^ there was insufficient evidence to recommend for or against screening. In our meta-analysis, only 2 trials^27,28^ began recruitment of patients after ERSPC and the Prostate, Lung, Colorectal, and Ovarian Cancer Screening Trial had published their results (eFigure 1 in the Supplement). Although these 2 trials^27,28^ reported results similar to our pooled results, it is possible that decision aids with new, updated evidence summaries may have more benefit than earlier decision aids in which results were more uncertain. There is therefore a call for new trials with updated decision aids.^12^ In general, trustworthy decision aids require links to recent evidence-based summaries and clinical practice guidelines that carry out dynamic updating.^12,14^

## Conclusions

Randomized clinical trials provide moderate-quality evidence that decision aids are associated with a small reduction in decisional conflict, while low-quality evidence suggests that they are associated with an increase in knowledge but not with whether physicians and patients discuss prostate cancer screening or with men’s decision to undergo or not undergo prostate cancer screening. The available evidence does not provide a compelling rationale for clinicians to use existing decision aids to facilitate shared decision-making in their discussions with men considering undergoing prostate cancer screening. Future decision aids should include provision for continuous updating and not only provide education to patients but also promote shared decision-making in the patient-physician encounter.

## Notes

Supplement. **eAppendix 1.** Search Strategies **eAppendix 2.** Case Examples of Definitions Used to Characterize Interventions **eAppendix 3.** Modified Version of Cochrane Risk of Bias Tool **eAppendix 4.** Modified Version of International Patient Decision Aid Standards Instrument (IPDASi v3) **eFigure 1.** Patient Recruitment Periods of the Included Decision Aid Trials in Comparison to the Publication of the Major Trials of Prostate Cancer Screening **eFigure 2.** Risk of Bias Evaluation Summary **eTable 1.** Summary of Decision Aids Used in Eligible Studies **eFigure 3.** The International Patient Decision Aid Standards Instrument Rating for Screening (IPDASi v3) Evaluation of Decision Aids **eFigure 4.** Pooled Analysis of Prostate Cancer Screening Long Term Knowledge **eTable 2.** Subgroup Analysis of Pooled Outcomes **eTable 3.** Excluded Studies in Alphabetical Order, With Reasons for Exclusion
