# Supplementary material for: Decision Aids for Prostate Cancer Screening Choice: A Systematic Review and Meta-analysis
Source: JAMA Intern Med. 2019 Jun 24;179(8):1072–82. doi: 10.1001/jamainternmed.2019.0763 (PMC6593633; doi:10.1001/jamainternmed.2019.0763)
Supplement: Supplement. — eAppendix 1. Search Strategies eAppendix 2. Case Examples of Definitions Used to Characterize Interventions eAppendix 3. Modified Version of Cochrane Risk of Bias Tool eAppendix 4. Modified Version of International Patient Decision Aid Standards Instrument (IPDASi v3) eFigure 1. Patient Recruitment Periods of the Included Decision Aid Trials in Comparison to the Publication of the Major Trials of Prostate Cancer Screening eFigure 2. Risk of Bias Evaluation Summary eTable 1. Summary of Decision Aids Used in Eligible Studies eFigure 3. The International Patient Decision Aid Standards Instrument Rating for Screening (IPDASi v3) Evaluation of Decision Aids eFigure 4. Pooled Analysis of Prostate Cancer Screening Long Term Knowledge eTable 2. Subgroup Analysis of Pooled Outcomes eTable 3. Excluded Studies in Alphabetical Order, With Reasons for Exclusion [file jamainternmed-179-1072-s001.pdf]

# Supplementary Online Content

Riikonen JM, Guyatt GH, Kilpeläinen TP, et al. Decision aids for prostate cancer screening choice: a systematic review and meta-analysis. *JAMA Intern Med.* Published online June 24, 2019. doi:10.1001/jamainternmed.2019.0763


**eAppendix 1.** Search Strategies
**eAppendix 2.** Case Examples of Definitions Used to Characterize Interventions
**eAppendix 3.** Modified Version of Cochrane Risk of Bias Tool
**eAppendix 4.** Modified Version of International Patient Decision Aid Standards Instrument (IPDASi v3)
**eFigure 1.** Patient Recruitment Periods of the Included Decision Aid Trials in Comparison to the Publication of the Major Trials of Prostate Cancer Screening
**eFigure 2.** Risk of Bias Evaluation Summary
**eTable 1.** Summary of Decision Aids Used in Eligible Studies
**eFigure 3.** The International Patient Decision Aid Standards Instrument Rating for Screening (IPDASi v3) Evaluation of Decision Aids
**eFigure 4.** Pooled Analysis of Prostate Cancer Screening Long Term Knowledge
**eTable 2.** Subgroup Analysis of Pooled Outcomes
**eTable 3.** Excluded Studies in Alphabetical Order, With Reasons for Exclusion


This supplementary material has been provided by the authors to give readers additional information about their work.

**eAppendix 1.** Search Strategies

1.1. Search strategy in Ovid MEDLINE

Database: OVID Medline Epub Ahead of Print, In-Process & Other Non-Indexed Citations, Ovid MEDLINE(R) Daily and Ovid MEDLINE(R) 1946 to Present
Search Strategy:
--------------------------------------------------------------------------------
1 exp Prostatic Neoplasms/ (114945)
2 (prostat$ adj3 (cancer$ or carcinoma$ or malignan$ or tumo?r$ or neoplas$ or metast$ or adeno$ or intraepithelial)).mp. (151028)
3 or/1-2 (151028)
4 exp Decision Making/ (178360)
5 Decision Support Systems, Clinical/ (6827)
6 exp decision support techniques/ (71485)
7 decision*.mp. (381799)
8 education*.mp. (899319)
9 "Early Detection of Cancer"/ or early detect*.mp. (69778)
10 (risk adj reduc*).mp. [mp=title, abstract, original title, name of substance word, subject heading word, floating sub-heading word, keyword heading word, protocol supplementary concept word, rare disease supplementary concept word, unique identifier, synonyms] (27640)
11 or/4-10 (1446995)
12 Prostate-Specific Antigen/ (23217)
13 PSA.mp. (31491)
14 prostate-specific.mp. (35303)
15 Mass Screening/ (94412)
16 screening.mp. (526209)
17 "Early Detection of Cancer"/ or early detect*.mp. (69778)
18 or/12-17 (612508)
19 3 and 11 and 18 (6406)
20 randomized controlled trial.pt. (465101)
21 controlled clinical trial.pt. (92523)
22 randomized.ab. (416937)
23 placebo.ab. (190560)
24 drug therapy.fs. (2031709)
25 randomly.ab. (293420)
26 trial.ab. (434077)
27 groups.ab. (1812893)
28 or/20-27 (4240617)
29 exp animals/ not humans.sh. (4476486)
30 28 not 29 (3666398)
31 19 and 30 (1805)
32 limit 19 to ("therapy (maximizes sensitivity)" or "therapy (maximizes specificity)" or "therapy (best balance of sensitivity and specificity)") (1682)

© 2019 Riikonen JM et al. *JAMA Intern Med*.

1.2. Search strategy in Ovid Embase

Database: Embase <1974 to 2018 June 19>
Search Strategy:

--------------------------------------------------------------------------------

1 exp prostate cancer/ (188754)
2 (prostat$ adj3 (cancer$ or carcinoma$ or malignan$ or tumo?r$ or neoplas$ or metast$ or adeno$ or intraepithelial)).mp. (238480)
3 1 or 2 (238480)
4 exp decision making/ (321508)
5 decision support system/ (19104)
6 decision*.mp. (607968)
7 education*.mp. (1168866)
8 early detect*.mp. (78053)
9 (risk adj reduc*).mp. [mp=title, abstract, heading word, drug trade name, original title, device manufacturer, drug manufacturer, device trade name, keyword, floating subheading word, candidate term word] (101219)
10 or/4-9 (1862547)
11 prostate specific antigen/ (50228)
12 PSA.mp. (58780)
13 prostate-specific.mp. (57503)
14 cancer screening/ (67992)
15 mass screening/ (54748)
16 screening/ or screening test/ (231910)
17 screening.mp. (920570)
18 early detect*.mp. (78053)
19 or/11-18 (1053852)
20 3 and 10 and 19 (9897)
21 clinical article/ (1921350)
22 exp clinical study/ (8614859)
23 clinical trial/ (969478)
24 controlled study/ (6031356)
25 randomized controlled trial/ (507171)
26 major clinical study/ (3140564)
27 double blind procedure/ (150957)
28 multicenter study/ (188317)
29 single blind procedure/ (31690)
30 phase 3 clinical trial/ (34593)
31 phase 4 clinical trial/ (3001)
32 crossover procedure/ (55897)
33 placebo/ (326824)
34 or/21-33 (12213796)
35 allocat$.mp. (142637)
36 assign$.mp. (341564)
37 blind$.mp. (441935)
38 (clinic$ adj25 (study or trial)).mp. (5648668)
39 compar$.mp. (7121723)
40 control$.mp. (9127665)

© 2019 Riikonen JM et al. *JAMA Intern Med*.

41 cross?over.mp. (88139)
42 factorial$.mp. (61683)
43 follow?up.mp. (42673)
44 placebo$.mp. (421608)
45 prospectiv$.mp. (1005004)
46 random$.mp. (1517078)
47 ((singl$ or doubl$ or trebl$ or tripl$) adj25 (blind$ or mask$)).mp. (283728)
48 trial.mp. (1897288)
49 (versus or vs).mp. (1846219)
50 or/35-49 (15415395)
51 34 and 50 (9518355)
52 exp animals/ or exp invertebrate/ or animal experiment/ or animal model/ or animal tissue/ or animal cell/ or nonhuman/ (26186623)
53 human/ or normal human/ or human cell/ (19815257)
54 52 and 53 (19766333)
55 52 not 54 (6420290)
56 51 not 55 (7366812)
57 20 and 56 (4494)
58 limit 20 to ("therapy (maximizes sensitivity)" or "therapy (maximizes specificity)" or "therapy (best balance of sensitivity and specificity)") (4213)

© 2019 Riikonen JM et al. *JAMA Intern Med*.

1.3. Search strategy in PsycINFO

Database: PsycINFO <1987 to June Week 2 2018>
Search Strategy:
--------------------------------------------------------------------------------

| | |
|---|---|
| 1 | prostat*.mp. [mp=title, abstract, heading word, table of contents, key concepts, original title, tests & measures] (3986) |
| 2 | prostate/ (1907) |
| 3 | 1 or 2 (3986) |
| 4 | exp decision making/ (85497) |
| 5 | decision support systems/ (2967) |
| 6 | decision*.mp. (180908) |
| 7 | education*.mp. (434165) |
| 8 | early detect*.mp. (5060) |
| 9 | (risk adj reduc*).mp. (5227) |
| 10 | or/4-9 (610400) |
| 11 | cancer screening/ (4144) |
| 12 | prostat*.mp. [mp=title, abstract, heading word, table of contents, key concepts, original title, tests & measures] (3986) |
| 13 | PSA.mp. (1037) |
| 14 | Health Screening/ or Screening/ (11580) |
| 15 | exp Screening Tests/ (5009) |
| 16 | screening.mp. (67567) |
| 17 | early detect*.mp. (5060) |
| 18 | or/11-17 (74975) |

© 2019 Riikonen JM et al. *JAMA Intern Med*.

1.4. Search strategy in Cochrane CENTRAL

CENTRAL (the trails database for the Cochrane Library)
Date Run: 20/06/18
Description:

| ID | Search |
|---|---|
| #1 | MeSH descriptor: [Prostatic Neoplasms] explode all trees (5382) |
| #2 | (prostat* near/3 (cancer$ or carcinoma$ or malignan$ or tumo?r$ or neoplas$ or metast$ or adeno$ or intraepithelial)) (11214) |
| #3 | #1 or #2 (11685) |
| #4 | MeSH descriptor: [Decision Making] explode all trees (4037) |
| #5 | MeSH descriptor: [Decision Support Techniques] explode all trees (3783) |
| #6 | MeSH descriptor: [Decision Support Systems, Clinical] explode all trees (403) |
| #7 | decision* (38798) |
| #8 | education* (68454) |
| #9 | MeSH descriptor: [Early Detection of Cancer] explode all trees (1139) |
| #10 | early detect* (15604) |
| #11 | risk near/1 reduc* (13455) |
| #12 | #4 or #5 or #6 or #7 or #8 or #9 or #10 or #11 (122996) |
| #13 | MeSH descriptor: [Prostate-Specific Antigen] explode all trees (1300) |
| #14 | PSA (4294) |
| #15 | prostate-specific (3566) |
| #16 | MeSH descriptor: [Mass Screening] explode all trees (5830) |
| #17 | screening (36095) |
| #18 | MeSH descriptor: [Early Detection of Cancer] explode all trees (1139) |
| #19 | early detect* (15604) |
| #20 | #13 or #14 or #15 or #16 or #17 or #18 or #19 (51415) |

© 2019 Riikonen JM et al. *JAMA Intern Med*.

1.5. Search strategy in CINAHL

CINAHL search:#

| | |
|---|---|
| S19 | S12 OR S13 OR S14 OR S15 OR S16 OR S17 OR S18 (95,547) |
| S18 | "early detect*" (9,054) |
| S17 | (MH "Early Detection of Cancer") (2,711) |
| S16 | "screening" (87,097) |
| S15 | (MH "Health Screening") OR (MH "Cancer Screening") (35,130) |
| S14 | "prostate-specific" (3,781) |
| S13 | "PSA" (2,569) |
| S12 | (MH "Prostate-Specific Antigen") (3,168) |
| S11 | S4 OR S5 OR S6 OR S7 OR S8 OR S9 OR S10 (571,297) |
| S10 | risk N1 reduc* (25,902) |
| S9 | "early detect*" (9,054) |
| S8 | (MH "Early Detection of Cancer") (2,711) |
| S7 | "education" (438,565) |
| S6 | "decision*" (121,229) |
| S5 | (MH "Decision Support Systems, Clinical") OR (MH "Decision-Making Support (Iowa NIC)") OR (MH "Decision Support Techniques") (4,740) |
| S4 | (MH "Decision Making+") (72,137) |
| S3 | S1 OR S2 (15,787) |
| S2 | (prostat* N3 (cancer$ or carcinoma$ or malignan$ or tumo?r$ or neoplas$ or metast$ or adeno$ or intraepithelial)) (11,112) |
| S1 | (MH "Prostatic Neoplasms+") (13,525) |

© 2019 Riikonen JM et al. *JAMA Intern Med*.

1.6. Search strategy in PubMed

PubMed
Search ((((((prostat*[Title]) AND decision*[Title/Abstract]) AND cancer[Title])) AND ((random*) OR trail*))) AND (((publisher[sb] OR inprocess[sb] OR pubmednotmedline[sb] OR pubstatusaheadofprint)))
Sort by: PublicationDate
Number of hits: 43

© 2019 Riikonen JM et al. *JAMA Intern Med*.

**eAppendix 2.** Case Examples of Definitions Used to Characterize Interventions

2.1. Information considered as usual care

Study: Wolf A, Nasser JF, Wolf AM, Schorling JB: The Impact of Informed Consent on Patient Interesting Prostate-Specific Antigen Screening. Arch Intern Med. 1996; 156: 1333-6.

*Control arm information was statement that "PSA is available and can sometimes detect early prostate cancer".*

Study: Frosch DL, Bhatnagar V, Tally S, Hamori CJ, Kaplan RM. Internet patient decision support: a randomized controlled trial comparing alternative approaches for men considering prostate cancer screening. Arch Intern Med. 2008; 168(4): 363-9.

*Participants assigned to the control group were provided links to public Websites on prostate cancer screening maintained by the American Cancer Society and the Centers for Disease Control and Prevention.*

2.2. Information considered as informative material (not decision aid)

Study: Stamatiou K, Skolarikos A, Heretis I, Papadimitriou V, Alevizos A, Ilias G, Karanasiou V, Mariolis A, Sofras F: Does educational printed material manage to change compliance with prostate cancer screening? World J Urol. 2008; 26: 365–73.

*"Screening means that men with no symptoms what-so ever are tested for prostate cancer. There are two ways of doing this: the digital rectal examination and the PSA measurement. Digital rectal examination is the simplest and least costly method but it is also the least effective when screening for prostate cancer. Used alone, it may miss almost 50% of cancers and is therefore inadequate for screening purposes. On the contrary, PSA measurement is much more effective, but it has also certain limits: even if the test shows an abnormally high level it does not necessarily mean that cancer is present. In fact, approximately 80% of men with one abnormal result do not have prostate cancer. Combining a PSA test and digital rectal examination is a more valuable means of screening than PSA measurement alone, and can identify 87% of prostate cancers"*

2.3. Decision aid comparing another decision aid

Study: Williams RM, Davis KM, Luta G, Edmond SN, Dorfman CS, Schwartz MD, Lynch J, Ahaghotu C, Taylor KL: Fostering Informed Decisions: Impact of a Decision Aid Among Men Registered to Undergo Mass Screening for Prostate Cancer in a Randomized Controlled Trial. Patient Educ Couns. 2013; 91(3): 329-36.

*Decision aid booklet—We adapted content from the Centers for Disease Control and Prevention's PCS educational tool. The booklet requires 20 minutes to read and includes information on the leading causes of death among men, the accuracy of the prostate-specific antigen test, PCS guidelines, and PCa diagnostic*

*procedures and treatments. The values clarification section is a 10-item adapted tool in which men indicate whether the items 'sound like you.' Five items indicate leaning towards screening and five items indicate leaning away from screening. We conducted focus groups and usability testing, which included a review by a plain language specialist to ensure that the DA did not exceed an 8th grade reading level. The end product was a 24-page color booklet entitled Prostate Cancer Screening: Making an Informed Decisio*

*Usual Care booklet—The UC booklet was the National Cancer Institute's "Questions and Answers About the Prostate Specific Antigen Test." This 3-page fact sheet requires 5 minutes to read and presented information in a Q&A format, who is recommended for testing, how to interpret results, and the limitations of testing. Given the differences in length, the UC booklet included less detail than the DA (e.g., it contained little information about the prostate, treatment options, and had no values clarification tool).*

Study: Davis SN, Sutton SK, Vadaparampil ST, Meade CD, Rivers BM ,Patel MV, Torres-Roca JF, Heysek RV, Spiess P, Pow-Sang J, Jacobsen PB, Gwede CK: Informed decision making among first-degree relatives of prostate cancer survivors: A pilot randomized trial. Contemporary Clinical Trials 2014; 39: 327-34.

*Seventy-eight Black and White first-degree relatives (FDRs) were randomized to one of two decision aid groups; 39 to a FDR-targeted decision aid and 39 to a general decision aid.*

*General intervention—consisted of the Centers for Disease Control and Prevention (CDC) PCa IDM booklet titled "Prostate cancer screening: Decision guide".*

*FDR targeted intervention—consisted of the CDC PCa IDM booklet and FDR-targeted decision aid DVD titled "Deciding about PC screening: A family matter".*

© 2019 Riikonen JM et al. *JAMA Intern Med*.

**eAppendix 3.** Modified Version of Cochrane Risk of Bias Tool

Modified from Guyatt GH & Busse JW. Modification of Cochrane Tool to Assess Risk of Bias in Randomized Trials. Available at https://www.evidencepartners.com/resources/methodological-resources/ (accessed 6 February 2019)

1) **Randomization**

a) **Was the allocation sequence adequately generated?**

| Definitely yes (low risk of bias) | Probably yes | Probably no | Definitely no (high risk of bias) |
|---|---|---|---|

The use of a random component should be sufficient for adequate sequence generation. This could be achieved by allocating interventions using methods such as repeated coin-tossing, throwing dice or dealing previously shuffled cards. If the allocation was by telephone or Internet, the randomization was done through a computer system.
Examples of low risk of bias: Referring to a random number table; Using a computer random number generator; Coin tossing; Shuffling cards or envelopes; Throwing dice; Drawing of lots; Minimization with or without a random element.

Examples of high risk of bias: Sequence generated by odd or even date of birth; Sequence generated by some rule based on date (or day) of admission; Sequence generated by some rule based on hospital or clinic record number; Allocation by judgement of the clinician; Allocation by preference of the participant; Allocation based on the results of a laboratory test or a series of tests; Allocation by availability of the intervention.

If they say “randomized” and give no more information regarding sequence generation, the process was probably low risk of bias, so, answer “Probably yes”.

b) **Was allocation adequately concealed?**

| Definitely yes (low risk of bias) | Probably yes | Probably no | Definitely no (high risk of bias) |
|---|---|---|---|

Examples of possible low risk of bias: Sequentially numbered drug containers of identical appearance; Sequentially numbered, opaque, sealed envelopes.

Examples of high risk of bias allocation generation techniques: Using an open random allocation schedule (e.g. a list of random numbers); Assignment envelopes were used without appropriate safeguards (e.g. if

© 2019 Riikonen JM et al. *JAMA Intern Med*.

envelopes were unsealed or non-opaque or not sequentially numbered); Alternation or rotation; Date of birth; Case record number; Any other explicitly unconcealed procedure.

Cochrane instructions:

- Use of telephone, web-based, independent research assistant, or pharmacy-controlled randomization → Central randomization
- Allocation by minimization → Central randomization
- Use of envelopes but at least one of the 3 descriptors or an equivalent (sequentially numbered, opaque, sealed) missing → Envelopes, other
- Use of a list of random numbers, a randomization table → Open random allocation schedule
- Use of alternation, rotation, date of birth, day of the week, or case record number → Quasi-randomized
- Explicitly described as concealed but no concealment method described → Concealed, no method described
- Explicitly described as not concealed → Not concealed
- No mention of a concealment method or of concealment at all → Not reported

Examples of low risk of bias allocation concealment techniques: Central allocation (including telephone, web-based, and pharmacy-controlled, randomization);

If they say “randomized” and give no more information regarding allocation concealment, the process was probably high risk of bias, so, answer “Probably no”.

2) **Blinding. Was knowledge of the allocated interventions adequately prevented?**

**a) Were data collectors blinded?**

| Definitely yes (low risk of bias) | Probably yes | Probably no | Definitely no (high risk of bias) |
|---|---|---|---|

If questionnaires were self-administered, patients were the data collectors. If questionnaires were interviewer-administered, the interviewers were the data collectors. Data collectors are those who abstracted the data from the medical records.
If the questionnaire were self- or interviewer-administered, you only answer “Definitely yes” or “Probably yes” if there was indication that the patients or the interviewers did not know that the information they got was decision aid. Otherwise it is “Definitely no” or “Probably no”.
For data collectors for abstracting the data from the medical records answer “Probably yes” unless there is some specific indication otherwise.

**b) Were data analysts blinded?**

| Definitely yes | Probably yes | Probably no | Definitely no |
|---|---|---|---|

© 2019 Riikonen JM et al. *JAMA Intern Med*.

(low risk of bias) (high risk of bias)

Answer “Probably no” unless there is some specific indication implying that data analysts were blinded.

**3) Missing data:**

**a) Screening choice**

**b) Other outcomes**

| Definitely yes | Probably yes | Probably no | Definitely no |
|---|---|---|---|
| (low risk of bias) | | | (high risk of bias) |

Risk of bias was considered high if more than 5% of data was missing.

© 2019 Riikonen JM et al. *JAMA Intern Med*.

**eAppendix 4.** Modified Version of International Patient Decision Aid Standards Instrument (IPDASi v3)

**1. SCREENING AIM: The decision support technology describes what the test is designed to measure.**
*We answered "yes" if the use of test informed by decision support technology was designed to measure whether man has prostate cancer.*

**2. TRUE POSITIVE: The decision support technology includes information about the chances of having a true positive test result.**
*We answered "yes" if the decision support technology included quantitative information about the chances of having prostate cancer when test was positive. If the decision support technology gave probability of positive test, and if it also gave the probability of having prostate cancer among those with positive test, we answered "yes".*

**3. TRUE NEGATIVE: The decision support technology includes information about the chances of having a true negative test result.**
*We answered "yes" if the decision support technology included quantitative information about the chances of not having prostate cancer when test was negative. If the decision support technology gave probability of negative test, and if it also gave the probability of not having prostate cancer among those with negative test, we answered "yes".*

**4. FALSE POSITIVE: The decision support technology includes information about the chances of having a false positive test result.**
*We answered "yes" if the decision support technology included quantitative information about the chances of not having prostate cancer when test was positive. If the decision support technology gave probability of positive test, and if it also gave the probability of not having prostate cancer among those with positive test, we answered "yes".*

**5. FALSE NEGATIVE: The decision support technology includes information about the chances of having a false negative test result.**
*We answered "yes" if the decision support technology included quantitative information about the chances of having prostate cancer when test was negative. If the decision support technology gave probability of negative test, and if it also gave the probability of having prostate cancer among those with negative test, we answered "yes".*

**6. NEXT STEPS IF POSITIVE: If the test detects the condition or problem, the decision support technology describes the next steps typically taken.**
*We rated as "yes" if the decision support technology informed that typical steps after positive test include repeating the test and referral to urologist if still positive or direct referral to urologist for further examination, which typically includes prostate biopsy. If prostate biopsies were not mentioned, we rated as "no".*

© 2019 Riikonen JM et al. *JAMA Intern Med*.

**7. NEXT STEPS IF NEGATIVE: The decision support technology describes the next steps if the condition or problem is not detected.**
*We rated as "yes" if the decision support technology informed that typical steps include either repeating the test at some time in the future, or no planned testing in future.*

***8. CHANCES OF DISEASE: The decision support technology describes the chances that the disease is detected with and without the use of the test.***
*We answered "yes" if the decision support technology described the chances that prostate cancer was detected with and without the use of test properties (=screening) or impact of test on outcomes.*

**9-10. The decision support technology presents the consequences of screening versus not screening on the outcomes of interest.**

- **9. IMPACT ON MORTALITY:** *We answered "yes" if the decision support technology described the screening effect on overall or prostate cancer specific mortality.*

- **10. HARMS OF SCREENING:** *We answered "yes" if the decision support technology described the chances that screening may lead to erectile dysfunction, urinary incontinence, and bowel problems. If 2/3 were mentioned, we gave points.*

© 2019 Riikonen JM et al. *JAMA Intern Med*.

**eFigure 1.** Patient Recruitment Periods of the Included Decision Aid Trials in Comparison to the Publication of the Major Trials of Prostate Cancer Screening

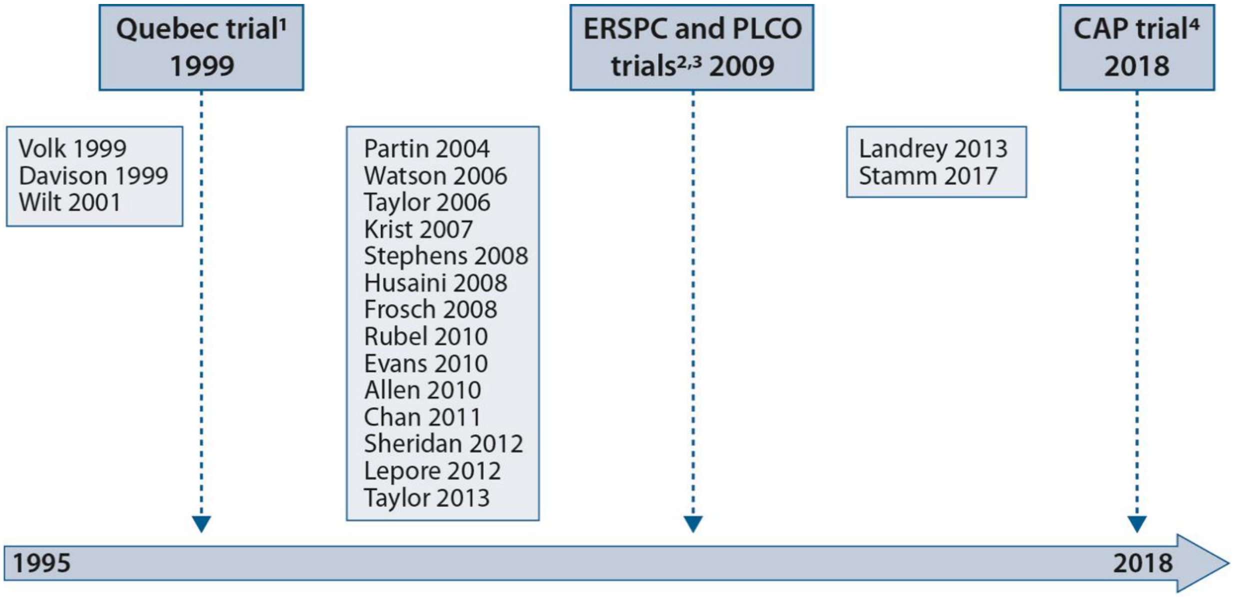



**References:**
1. Labrie et al. Prostate 1999; 2. Schröder et al. N Engl J Med 2009; 3. Andriole et al. N Engl J Med 2009; 4. Martin et al. JAMA 2018


© 2019 Riikonen JM et al. *JAMA Intern Med*.

**eFigure 2.** Risk of Bias Evaluation Summary

| | Randomization | | Blinding | | Missing data | | | | | Overall risk of bias |
|---|---|---|---|---|---|---|---|---|---|---|
| | Sequence generation | Allocation concealment | Data collectors | Data analysts | Knowledge | Decisional conflict | Screening discussions | Screening choice | Satisfaction with decision | |
| Stamm 2017 | + | – | – | + | – | | | – | | High |
| Landrey 2013 | + | – | + | – | – | | – | – | | High |
| Taylor 2013 | + | + | – | – | – | – | | – | – | High |
| Lepore 2012 | + | + | + | – | – | – | – | + | | High |
| Sheridan 2012 | + | + | + | – | + | | + | + | | **Low** |
| Chan 2011* | + | – | + | – | + | | | | | **Low** |
| Allen 2010* | + | – | – | – | – | – | | | | High |
| Evans 2010 | + | + | – | – | – | – | | + | | High |
| Rubel 2010 | + | + | – | – | + | + | | | | **Low** |
| Frosch 2008 | + | + | + | – | – | – | | – | | High |
| Husaini 2008* | + | – | – | – | + | | | + | | High |
| Stephens 2008 | + | – | + | – | + | + | | | | **Low** |
| Krist 2007 | + | + | – | – | + | + | + | – | | High |
| Taylor 2006 | + | – | – | – | – | – | | | – | High |
| Watson 2006 | + | – | – | – | – | | | | | High |
| Partin 2004 | + | + | + | – | – | | – | – | | High |
| Wilt 2001 | + | + | + | – | + | | + | + | | **Low** |
| Davison 1999 | + | – | – | – | | + | | + | | High |
| Volk 1999 | + | – | – | – | – | | | – | – | High |

*a cluster randomized trial

© 2019 Riikonen JM et al. *JAMA Intern Med*.

**eTable 1.** Summary of Decision Aids Used in Eligible Studies

| Ref | Type of decision aid | Name of decision aid | How did we identify the decision aids | Evaluated decision aid | Provision of continuous updating |
|---|---|---|---|---|---|
| Stamm 2017 | Decision aid and script shared decision making | Not reported | Not found | | |
| Landrey 2013 | Leaflet print | “Prostate cancer screening” | From original article | Leaflet print | |
| Taylor 2013 | 1) Booklet print*<br>2)Computer-based* | 1) “Prostate Cancer Screening. Making an Informed Decision”<br>2) “Prostate Cancer Screening. Making the Best Choice” | 1) From electrical search<br>2) Found by not able to evaluate | 1) Booklet print | 2) Yes, 2014 |
| Lepore 2012 | Telephone education<br>Booklet print | Not reported<br>“Prostate Cancer: Your Life – You Decide” | From original article<br>Not found | Telephone education | |
| Sheridan 2012 | Video<br>Individual education<br>Leaflet print | Not reported.<br>Not reported<br>“You Decide. Deciding Whether or Not to Get a PSA Test for Prostate Cancer” | Described in article<br>Described in article<br>From original article | Video<br>Coaching<br>Leaflet print | |
| Chan 2011 | Group education: video, booklet print, script, slides | ”Men’s Guide” booklet print<br>Others not reported | Not found | | |
| Allen 2010 | Computer-based | “Take the Wheel” | Not found | | |
| Evans 2010 | 1) Computer-based<br>2) Booklet print | 1) ”Prosdex”<br>2) Not reported | 1) From electrical search<br>2) Not found | 1) Computer-based | |
| Rubel 2010 | Booklet print | Not reported | Not available | | |
| Frosch 2008 | Computer-based<br>1) Chronic disease trajectory model<br>2) Traditional<br>3) Combination of 1 and 2 | 1-3) Not reported | 1-3) Not found | | |
| Husaini 2008 | Group education: video, leaflet, teaching session | Not reported | Not found | | |
| Stephens 2008 | Booklet print | ”Prostate Cancer Screening. A Decision Guide for African Americans” | From electronic search | Booklet print | |
| Krist 2007 | 1) Booklet print*<br>2) Computer-based* | 1 ”Should you get a PSA test? A Patient-Doctor decision”<br>2) ”Should you get a PSA test? A Patient-Doctor decision” | 1) From author<br>2) Not found | 1) Booklet print | 1) Yes, 2017 |
| Taylor 2006 | 1) Video<br>2) Booklet print | 1) Not reported<br>2) “The Right Decision Is Yours. A Guide to Prostate Cancer Check-ups” | 1) Not found<br>2) From electronic search | 2) Booklet print | |
| Watson 2006 | Leaflet print | “PSA Testing for Prostate Cancer” | From original article | Leaflet print | |
| Partin 2004 | 1) Booklet print<br>2) Video** | 1) “The PSA test for prostate cancer. Is it right for me?”<br>2) “The PSA Decision: What YOU Need to Know” | 1) From author<br>2) From author | 1) Booklet print<br>2) Video** | |
| Wilt 2001 | Leaflet print | ”Early Prostate Cancer” | From original article | Leaflet print | |
| Davison 1999 | Individual education: verbal and print | Not reported | Not available | | |
| Volk 1999 | Video**<br>Leaflet print | “The PSA Decision: What YOU Need to Know”<br>Not reported | From other author (Partin 2004)<br>Not found | Video** | |

* Identical information between study groups., ** Same video used in Partin 2004 and Volk 1999 studies.
Numbers 1, 2 and 3 mean that decision aids were used in different groups. If there is no number, there was one decision aid group.

© 2019 Riikonen JM et al. *JAMA Intern Med*.

**eFigure 3.** The International Patient Decision Aid Standards Instrument Rating for Screening (IPDASi v3) Evaluation of Decision Aids

| | Screening aim | True positive | True negative | False positive | False negative | Next steps if positive | Next steps if negative | Chance of disease | Mortality | Harms | **SCORE** |
|---|---|---|---|---|---|---|---|---|---|---|---|
| Landrey 2013 | + | – | – | – | – | – | – | – | – | + | 2 |
| Taylor 2013 | + | + | + | + | + | + | + | – | + | + | 9 |
| Lepore 2012 | + | – | – | – | – | – | – | – | + | – | 2 |
| Sheridan 2012 | + | – | – | – | – | + | – | – | + | + | 4 |
| Evans 2010 | + | + | + | + | + | + | + | + | + | + | 10 |
| Stephens 2008 | + | + | + | + | – | + | – | – | + | + | 7 |
| Krist 2007 | + | + | + | + | + | + | – | + | + | + | 9 |
| Taylor 2006 | + | – | – | – | – | – | – | – | + | – | 2 |
| Watson 2006 | + | + | – | + | – | + | + | – | + | + | 7 |
| Partin 2004 (Print) | + | – | – | – | – | + | – | – | + | + | 4 |
| Partin 2004 (Video*) | + | + | – | – | – | + | – | + | + | + | 6 |
| Wilt 2001 | + | + | – | + | – | – | – | – | + | + | 5 |
| Volk 1999* | + | + | – | – | – | + | – | + | + | + | 6 |

*Same video used in Partin 2004 and Volk 1999 studies

© 2019 Riikonen JM et al. *JAMA Intern Med*.

**eFigure 4.** Pooled Analysis of Prostate Cancer Screening Long Term Knowledge

**Knowledge – long term (adjusted)**

| Study | Decision aid Mean/SE | No decision aid Mean/SE | Weight (%) | Mean difference (95 % CI) |
|---|---|---|---|---|
| Husaini 2008 | 5.33/1.39 | 4.67/1.98 | 34 | 0.67 (-4.07, 5.41) |
| Allen 2010 | 10.00/3.39 | 4.00/2.02 | 25 | 6.00 (-1.74, 13.74) |
| Taylor 2013* | 12.51/0.75 | 3.33/0.98 | 41 | 9.17 (6.76, 11.59) |
| Random effects, p=0.007 for heterogeneity, $I^2$=80% | | | | 5.47 (-0.52, 11.45) |

*pooled result from multi-arms

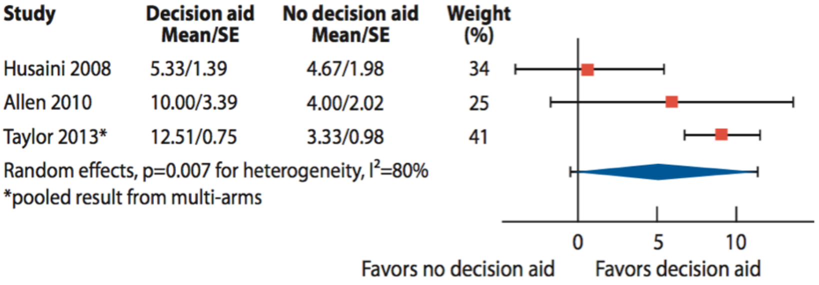


© 2019 Riikonen JM et al. *JAMA Intern Med*.

**eTable 2.** Subgroup Analysis of Pooled Outcomes

| **Predictor** | **Subgroup category** | **Sample size** | **Estimate** | ***P* Value** |
|---|---|---|---|---|
| Knowledge, short term | Allocation concealment | 1=low risk, n=2 | 12.47 (-14.52, 39.47) | .72 |
| | | 0=high risk, n=7 | 17.28 (2.67, 31.90) | |
| | Data collector blinding | 1=low risk, n=6 | 11.62 (-2.70, 25.93) | .23 |
| | | 0=high risk, n=3 | 25.15 (5.11, 45.19) | |
| | Missing data | 1=low risk, n=3 | 16.75 (-5.75, 39.24) | .95 |
| | | 0=No, n=6 | 15.92 (0.03, 31.80) | |
| | Adjusted from baseline | 1=Yes, n=5 | 15.05 (-2.27, 32.38) | .82 |
| | | 0=high risk, n=4 | 17.63 (-1.79, 37.05) | |
| Knowledge, long term | Allocation concealment | 1=low risk, n=1 | 9.17 (-31.31, 49.66) | .37 |
| | | 0=high risk, n=2 | 2.93 (-30.56, 36.42) | |
| | Data collector blinding | 1=low risk, n=0 | / | |
| | | 0=high risk, n=3 | / | |
| | Missing data | 1=low risk, n=1 | 0.67 (-26.54, 27.88) | .22 |
| | | 0=high risk, n=2 | 7.96 (-11.64, 27.56) | |
| Decisional conflict, take the longest follow up | Allocation concealment | 1=low risk, n=3 | -3.39 (-8.49, 1.70) | .56 |
| | | 0=high risk, n=4 | -5.05 (-9.46, -0.63) | |
| | Data collector blinding | 1=low risk, n=3 | -4.15 (-9.44, 1.14) | .91 |
| | | 0=high risk, n=4 | -4.48 (-9.06, 0.10) | |
| | Missing data | 1=low risk, n=4 | -4.69 (-9.95, 0.57) | .62 |
| | | 0=high risk, n=2 | -2.95 (-10.39, 4.49) | |

© 2019 Riikonen JM et al. *JAMA Intern Med*.

**eTable 3.** Excluded Studies in Alphabetical Order, With Reasons for Exclusion

| **Study** | **Reason for exclusion** |
|---|---|
| Adarkwah CC, Kuss K, Donner-Banzhoff N, Semjonow A, Hense HW, Simbrich A. Boon or bane? Arriba PSA: a new decision aid to support PSA-screening counselling. *Eur J Gen Pract.* 2017;23(1):148. | Ineligible decision aid intervention |
| Berglund G, Nilsson S, Nordin K. Intention to test for prostate cancer. *Eur J Cancer.* 2005;41(7):990-997. | Ineligible outcomes |
| Braun KL, Thomas WL Jr, Domingo JL, et al. Reducing cancer screening disparities in medicare beneficiaries through cancer patient navigation. *J Am Geriatr Soc.* 2015;63(2):365-370. | Ineligible decision aid intervention |
| Davis SN, Sutton SK, Vadaparampil ST, et al. Informed decision making among first-degree relatives of prostate cancer survivors: a pilot randomized trial. *Contemp Clin Trials.* 2014;39(2):327-334. | Ineligible control group |
| Doorenbos AZ, Jacobsen C, Corpuz R, Forquera R, Buchwald D. A randomized controlled calendar mail-out to increase cancer screening among urban American Indian and Alaska Native patients. *J Cancer Educ.* 2011;26(3):549-554. | Ineligible decision aid intervention |
| Ellison GL, Weinrich SP, Lou M, Xu H, Powell IJ, Baquet CR. A randomized trial comparing web-based decision aids on prostate cancer knowledge for African-American men. *J Natl Med Assoc.* 2008;100(10):1139-1145. | Ineligible control group |
| Frosch DL, Kaplan RM, Felitti VJ. Evaluation of two methods to facilitate shared decision making for men considering the prostate-specific antigen test. *J Gen Intern Med.* 2001;16(6):391-398. | Ineligible control group |
| Frosch DL, Kaplan RM, Felitti VJ. A randomized controlled trial comparing internet and video to facilitate patient education for men considering the prostate specific antigen test. *J Gen Intern Med.* 2003;18(10):781-787. | Ineligible control group |
| Gattellari M, Ward JE. Does evidence-based information about screening for prostate cancer enhance consumer decision-making? A randomised controlled trial. *J Med Screen.* 2003;10(1):27-39. | Ineligible control group |
| Gattellari M, Ward JE. A community-based randomised controlled trial of three different educational resources for men about prostate cancer screening. *Patient Educ Couns.* 2005;57(2):168-182. | Ineligible control group |
| Holt CL, Le D, Slade JL, et al. Can Women Facilitate Men's Prostate Cancer Screening Informed Decision-Making? The M-PACT Trial. *J Health Commun.* 2017;22(12):964-973. | Ineligible control group |
| Holt CL, Wynn TA, Litaker MS, Southward P, Jeames S, Schulz E. A comparison of a spiritually based and non-spiritually based educational intervention for informed decision making for prostate | Ineligible control group |

© 2019 Riikonen JM et al. *JAMA Intern Med*.

| | |
|---|---|
| cancer screening among church-attending African-American men. *Urol Nurs.* 2009;29(4):249-258. | |
| Holt CL, Le D, Saunders DR, et al. Informed decision-making and satisfaction with a church-based men's health workshop series for African-American men: men-only vs. mixed-gender format. *J Cancer Educ.* 2015;30(3):530-534. | Ineligible control group |
| Ilic D, Egberts K, McKenzie JE, Risbridger G, Green S. Informing men about prostate cancer screening: a randomized controlled trial of patient education materials. *J Gen Intern Med.* 2008;23(4):466-471. | Ineligible control group |
| Kripalani S, Sharma J, Justice E, et al. Low-literacy interventions to promote discussion of prostate cancer: a randomized controlled trial. *Am J Prev Med.* 2007;33(2):83-90. | Ineligible decision aid intervention |
| Layton B. Effects of a web-based decision aid on African American men's prostate screening knowledge and behaviour. Minneapolis, Minnesota: Walden University, 2012. [dissertation] | Ineligible control group |
| Lewis CL, Adams J, Tai-Seale M, et al. A randomized controlled effectiveness trial for PSA screening decision support interventions in two primary care settings. *J Gen Intern Med.* 2015;30(6):810-816. | Low decision aid adherence |
| Molazem Z, Ebadi M, Khademian M, Zare R. Effects of an Educational Program for Prostate Cancer Prevention on knowledge and PSA Testing in Men Over 50 Years old in Community Areas of Shiraz in 2016. *Asian Pac J Cancer Prev.* 2018;19(3):633-637. | Ineligible decision aid intervention |
| Myers RE, Chodak GW, Wolf TA, et al. Adherence by African American men to prostate cancer education and early detection. *Cancer.* 1999;86(1):88-104. | Ineligible control group |
| Myers RE, Daskalakis C, Cocroft J, et al. Preparing African-American men in community primary care practices to decide whether or not to have prostate cancer screening. *J Natl Med Assoc.* 2005;97(8):1143-1154. | Ineligible control group |
| Myers RE, Daskalakis C, Kunkel EJ, et al. Mediated decision support in prostate cancer screening: a randomized controlled trial of decision counseling. *Patient Educ Couns.* 2011;83(2):240-246. | Ineligible control group |
| Pignone MP, Howard K, Brenner AT, et al. Comparing three techniques for eliciting patient values fordecision making about prostate specific antigen screening: arandomized controlled trial. *JAMA Intern Med.* 2013;173(5):362-368. | Ineligible control group |
| Royse D, Dignan M. Improving cancer knowledge and screening awareness: test of a telephone interviewer intervention. *J Cancer Educ.* 2009;24(4):315-318. | Ineligible decision aid intervention |
| Rupper RW, Lee Y, Marsh S, Alder SC, Burt RW. The influence of functional impairment on cancer screening rates in a navigation intervention for underserved American Indians. AGS 2013 Annual Meeting;A106:S53. | Ineligible control group |

© 2019 Riikonen JM et al. *JAMA Intern Med*.

| | |
|---|---|
| Salkeld G, Cunich M, Dowie J, Howard K, Mann G, Patel M. The role of personalized choice: a randomized controlled trial of an online decision aid for prostate cancer screening. *J Urol.* 2013;189:e511. | Ineligible control group |
| Saver BG, Mazor KM, Luckmann R, et al. Persuasive interventions for controversial cancer screening recommendations: testing a novel approach to help patients make evidence-based decisions. *Ann Fam Med.* 2017;15(1):48-55. | Ineligible control group |
| Schapira MM, VanRuiswyk J. The effect of an illustrated pamphlet decision-aid on the use of prostate cancer screening tests. *J Fam Pract.* 2000;49(5):418-424. | Ineligible control group |
| Sheridan SL, Sutkowi-Hemstreet A, Barcaly C et al. A comparative effectiveness trial of alternate formats for presenting benefits and harms information for low-value screening services. A randomized clinical trial. *JAMA Intern Med.* 2016;176(1):31-41. | Ineligible control group |
| Stamatiou K, Skolarikos A, Heretis I, et al. Does educational printed material manage to change compliance with prostate cancer screening? *World J Urol.* 2008;26(4):365-373. | Ineligible decision aid intervention |
| Thomas R, Glasziou P, Rychetnik L2 Mackenzie G, Gardiner R, Doust J. Deliberative democracy and cancer screening consent: a randomised control trial of the effect of a community jury on men's knowledge about and intentions to participate in PSA screening. *BMJ Open.* 2014;4(12):e005691. | Ineligible control group |
| Tran VT, Kisseleva-Romanova E, Rigal L, Falcoff H. Impact of a printed decision aid on patients' intention to undergo prostate cancer screening: a multicentre, pragmatic randomised controlled trial in primary care. *Br J Gen Pract.* 2015;65(634):e295-304. | Ineligible outcomes |
| Volk RJ, Jibaja-Weiss ML, Hawley ST, et al. Entertainment education for prostate cancer screening: a randomized trial among primary care patients with low health literacy. *Patient Educ Couns.* 2008;73(3):482-489. | Ineligible control group |
| Watts KJ, Meiser B, Wakefield CE, et al. Online prostate cancer screening decision aid for at-risk men: a randomized trial. *Health Psychol.* 2014;33(9):986-997. | Ineligible control group |
| Weiner AB, Tsai KP, Keeter MK, et al. The Influence of decision aids on prostate cancer screening preferences: A randomized survey study. *J Urol.* 2018;200(5):1048-1055. | Ineligible control group |
| Weinrich SP, Seger R, Curtsinger T, Pumphrey G, NeSmith EG, Weinrich MC. Impact of pretest on posttest knowledge scores with a Solomon Four research design. *Cancer Nurs.* 2007;30(5):E16-28. | Ineligible control group |
| Wilkes MS, Day FC, Srinivasan M, et al. Pairing physician education with patient activation to improve shared decisions in prostate cancer screening: a cluster randomized controlled trial. *Ann Fam Med.* 2013;11(4):324-334. | Ineligible outcomes |

| | |
|---|---|
| Williams RM, Davis KM, Luta G, et al. Fostering informed decisions: a randomized controlled trial assessing the impact of a decision aid among men registered to undergo mass screening for prostate cancer. *Patient Educ Couns.* 2013;91(3):329-336. | Ineligible control group |
| Wolf AM, Nasser JF, Wolf AM, Schorling JB. The impact of informed consent on patient interest in prostate-specific antigen screening. *Arch Intern Med.* 1996;156(12):1333-1336. | Ineligible outcomes |
| Zare M, Ghodsbin F, Jahanbin I, Ariafar A, Keshavarzi S, Izadi T. The Effect of Health Belief Model-Based Education on Knowledge and Prostate Cancer Screening Behaviors: A Randomized Controlled Trial. *Int J Community Based Nurs Midwifery.* 2016;4(1):57-68. | Ineligible outcomes (not able to meta-analyze the data) |

© 2019 Riikonen JM et al. *JAMA Intern Med*.
